# Hyperuricemia Causes Pancreatic β-Cell Death and Dysfunction through NF-κB Signaling Pathway

**DOI:** 10.1371/journal.pone.0078284

**Published:** 2013-10-25

**Authors:** Lu Jia, Jing Xing, Ying Ding, Yachen Shen, Xuhui Shi, Wei Ren, Meng Wan, Jianjin Guo, Shujing Zheng, Yun Liu, Xiubin Liang, Dongming Su

**Affiliations:** 1 Center of Metabolic Disease Research, Nanjing Medical University, Nanjing, Jiangsu Province, China; 2 Center of Cellular Therapy, The Second Affiliated Hospital of Nanjing Medical University, Nanjing, Jiangsu Province, China; 3 Department of Endocrinology and Metabolism, Shanghai Jiaotong University Affiliated Sixth People’s Hospital; Shanghai Diabetes Institute; and Shanghai Clinical Center of Diabetes, Shanghai, China; 4 Department of Pharmacy, Nanjing First Hospital, Nanjing Medical University, Nanjing, Jiangsu Province, China; Sun Yat-sen University Cancer Center, China

## Abstract

Accumulating clinical evidence suggests that hyperuricemia is associated with an increased risk of type 2 diabetes. However, it is still unclear whether elevated levels of uric acid can cause direct injury of pancreatic β-cells. In this study, we examined the effects of uric acid on β-cell viability and function. Uric acid solution or normal saline was administered intraperitoneally to mice daily for 4 weeks. Uric acid-treated mice exhibited significantly impaired glucose tolerance and lower insulin levels in response to glucose challenge than did control mice. However, there were no significant differences in insulin sensitivity between the two groups. In comparison to the islets in control mice, the islets in the uric acid–treated mice were markedly smaller in size and contained less insulin. Treatment of β-cells *in vitro* with uric acid activated the NF-κB signaling pathway through IκBα phosphorylation, resulting in upregulated inducible nitric oxide synthase (iNOS) expression and excessive nitric oxide (NO) production. Uric acid treatment also increased apoptosis and downregulated Bcl-2 expression in Min6 cells. In addition, a reduction in insulin secretion under glucose challenge was observed in the uric acid–treated mouse islets. These deleterious effects of uric acid on pancreatic β-cells were attenuated by benzbromarone, an inhibitor of uric acid transporters, NOS inhibitor L-NMMA, and Bay 11–7082, an NF-κB inhibitor. Further investigation indicated that uric acid suppressed levels of MafA protein through enhancing its degradation. Collectively, our data suggested that an elevated level of uric acid causes β-cell injury via the NF-κB-iNOS-NO signaling axis.

## Introduction

In the last few decades, the prevalence of hyperuricemia has been rapidly increasing worldwide [1], [2]. Meanwhile, a large body of evidence has established the association of elevated serum uric acid with various metabolic disorders, including gout, hypertension, atherosclerosis, renal diseases, and so on [3]. The question of whether hyperuricemia is associated with type 2 diabetes was raised about two decades ago. Recently, evidence has emerged from several large epidemiological studies which indicates that people with hyperuricemia are susceptible to type 2 diabetes [4], [5], [6]. However the causal mechanisms of hyperuricemia on the development of type 2 diabetes are still poorly determined.

Impaired β-cell survival and function are major contributors to the progression of diabetes. NF-κB activation and subsequent nitric oxide (NO) production by inducible nitric oxide synthase (iNOS) have been implicated in β-cell damage and death in both type 1 and type 2 diabetes [7], [8], [9]. The transcription factor NF-κB is activated by a variety of stimuli, including cellular stress and proinflammatory cytokines. Activation of NF-κB predominantly occurs via the release of the p50/p65 heterodimer from the inhibitor of κB (IκB) complex in the cytosol of the cells. This step is induced by IκB kinase (IKK)-mediated phosphorylation of inhibitory molecules, including IκBα. When released from IκB, the p50/p65 dimer translocates to the cell nucleus and regulates downstream gene expression. Inducible nitric oxide synthase (iNOS) is one of the major target genes of the NF-κB signaling pathway [10]. The expression of iNOS increases in β-cells of diabetic rodent models, leading to β-cell death. Conversely, silencing iNOS or the NF-κB gene protects against diabetes development in streptozotocin-treated mice and nonobese diabetic mice [11], [12].

Insulin biosynthesis and secretion by β-cells is finely regulated by various essential transcription factors, including MafA, PDX-1, and NeuroD, among others. The suppression of MafA leads to a marked reduction in insulin production [13]. Recent studies have demonstrated that dysfunctional MafA expression at both transcriptional and posttranslational levels leads to a loss of insulin gene expression [14].

In the current study, designed to examine the effects of uric acid on β-cell viability and function in the mouse, we found that uric acid impaired insulin secretion and survival of β-cells. We also demonstrated that uric acid increased NF-κB transcriptional activity but decreased MafA activity. We therefore chose to test the hypothesis that uric acid directly induces β-cell dysfunction, with the involvement of the NF-κB-iNOS-NO pathway and the transcription factor MafA.

## Materials and Methods

### Ethics Statement

Animals were treated humanely, using approved procedures in accordance with the guidelines of the Institutional Animal Care and Use Committee at Nanjing Medical University. The study was approved by the Experimental Animal Ethics Committee at the Nanjing Medical University.

### Reagents and Cell Culture

Unless otherwise stated, all chemical reagents were purchased from Sigma-Aldrich (Saint Louis, MO, USA). Min6 cells were obtained from ATCC (American Type Culture Collection, Manassas, USA) and maintained in 5 mM glucose DMEM (Hyclone, Logan, UT, USA), supplemented with 10% FBS, 50 µmol/L β-mercaptoethanol, 100 U/ml penicillin, and 0.1 mg/ml streptomycin in 5% CO_2_ at 37°C. Uric acid solution for cell treatments was prepared using prewarmed cell culture medium and passed through a 2.2 µm sterile filter, as previously described [15]. Antibodies to insulin, MafA and PDX-1 were purchased from Santa Cruz Biotechnology (Santa Cruz, CA, USA). Antibody to Bcl-2, phospho-IκBα (Ser32), IκBα, and NF-κB p65 were purchased from Cell Signaling Technology (Cell Signaling Technology, MA, USA).

### Islet Isolation

Islets were isolated from 8-week-old C57BL mice (Shanghai Experimental Animal Center of the Chinese Academy of Science, Shanghai, China) by collagenase digestion as previously described [16]. The islets were then cultured overnight in complete CMRL-1066 (Invitrogen, Carlsbad, CA, USA) with 10% FBS (Hyclone), containing 2 mM L-glutamine (Cambrex, Walkersville, MD, USA), 100 U/ml penicillin, 100 µg/ml streptomycin, and 5 mM glucose in 5% CO_2_ at 37°C.

### Glucose-stimulated Insulin Secretion Assay

Min6 cells or isolated mouse islets (eight islets per well) were seeded in 24-well plates and treated with uric acid for 24 h. After incubation for 1 h in glucose-free Krebs-Ringer bicarbonate (KRB) buffer (115 mM NaCl, 4.7 mM KCl, 1.2 mM MgSO_4_•7H_2_O, 1.2 mM KH_2_PO_4_, 20 mM NaHCO_3_, 16 mM HEPES, 2.56 mM CaCl_2,_ 0.2% BSA), the cells were treated for 1 h in KRB buffer with low (3.3 mmol/L) or high (16.7 mmol/L) concentrations of glucose [17]. After treatment, the supernatants were obtained for the determination of insulin concentration using radioimmunoassay as previously described [18], [19].

### Apoptosis Assay

Min6 or INS-1 cells were grown on glass coverslips in 24-well plates and incubated for 48 h in complete medium with either control, 5 mg/dL uric acid, Bay11–7082(5 µmol/L), L-NMMA(1 mmol/L), Benzbromarone(50 µmol/L), Uric acid(5 mg/dL), Uric acid(5 mg/dL)+Bay11–7082(5 µmol/L), Uric acid(5 mg/dL)+L-NMMA(1 mmol/L), or Uric acid(5 mg/dL)+Benzbromarone(50 µmol/L). Cells were then fixed and permeabilized and the TUNEL assay was performed according to the manufacturer’s instructions (In Situ Cell Death Detection Kit; Roche, Basel, Switzerland). Apoptosis was determined by TUNEL assay and by scoring cells displaying pyknotic nuclei, visualized by staining with the DNA-binding dye, Hoechst 33342 (Roche, Basel, Switzerland) [20], [21].

### RNA Isolation and RT-PCR Analysis

Total RNA was extracted from cultured cells and isolated islets using RNA isolator Total RNA Extraction Reagent (Vazyme Biotech, Nanjing, China). cDNA was synthesized from total RNA (1 µg) using PrimeScript® RT reagent Kit (Takara, Dalian, China) following the manufacturer’s instructions. RT-PCR was performed using SYBR® Premix Ex Taq™ (Takara, Dalian, China). Expression of iNOS was determined using RT-PCR analysis. Primers used to identify iNOS were: forward, 5′-CCCTTCCGAAGTTTCTGGCAGCAGCAGC-3′ and reverse, 5′-GGCTGTCAGAGCCTCGTGGCTTTGG-3′. β-actin was used as an input: forward, 5′-GCAAGTGCTTCTAGGCGGAC-3′ and reverse, 5′- AAGAAAGGGTGTAAAACGCAGC -3′.

### Western Blot Analysis

Cells were washed twice in ice-cold PBS, and then solubilized in RIPA lysis buffer (Vazyme Biotech, Nanjing, China). Samples containing equal amounts of protein were separated on 8–12% SDS-polyacrylamide gels. Proteins were then transferred to nitrocellulose membranes. Membranes were blocked in 5% non-fat milk for 1 h at room temperature. Proteins were immunoblotted with appropriate antibodies overnight at 4°C, followed by binding with peroxidase-labeled secondary antibodies for 1 h at room temperature. Immunoreactivity was detected by ECL reagents (MultiSciences Biotech, Hangzhou, China) using a Bio-Rad ChemiDoc™ XRS+ Universal HoodII machine.

### Nitrite Assay

Released NO was measured using a Griess Assay Kit (Beyotime, Shanghai, China) according to the manufacturer’s instructions.

### Animal Model

Eight-week-old male BALB/c mice (Shanghai Experimental Animal Center of the Chinese Academy of Science), weighing 18–22 g, were housed in the animal facility at the Experimental Animal Center of Nanjing Medical University with free access to food and water. Animals were randomly divided into control (n = 8) or hyperuricemia groups (n = 10). The mouse hyperuricemia model was generated by daily intraperitoneal injection of uric acid (250 mg/kg, Sigma) for 4 weeks [22], [23]. For the control group, normal saline was administered. At 2 weeks and 4 weeks, blood samples were harvested to test serum uric acid concentration using a Serum Uric Acid Detection Kit (Nanjing Jiancheng Bioengineering Institute, Nanjing, China). After 4 weeks of injections, mice were given glucose tolerance and insulin tolerance tests. Mice were killed and pancreases were fixed in 10% phosphate-buffered formalin followed by paraffin embedding.

### Glucose Tolerance Test

Mice were fasted overnight and injected intraperitoneally with 10% glucose solution (China Otsuka Pharmaceutical Co., Ltd., Tianjin, China) at 2 g/kg body weight. Blood glucose levels were measured at 0, 30, 60, 90, and 120 min using a Roche Accu-Chek Performa. For measuring serum insulin levels, blood samples were collected at 0 and 30 min. Serum insulin levels were detected using an Ultra Sensitive Mouse Insulin ELISA Kit (Crystal Chem, Downers Grove, IL, USA) [16], [24].

### Insulin Tolerance Test

After 6 h fasting, regular human insulin (0.75 U/kg) (Novo Nordisk, Beijing, China) was administered to mice intraperitoneally. Blood glucose levels were measured before insulin administration and then afterwards, every 15 min, for 120 min [16], [24].

### Immunohistochemistry Studies

Since the mouse pancreas presents in a mesenteric pattern, whose anatomic parts are impossible to be differentiated accurately, thus the whole mouse pancreas was subjected to histological analysis. Slides of 4 µm sections of pancreas from each group (control and 250 mg/kg uric acid) were stained for insulin using anti-insulin antibody (Santa Cruz, CA, USA). 20 islets from each group were randomly selected and used to calculate the area of islets and the intensity of insulin staining using Image-Pro Plus software. The immunohistochemical procedure and image analysis procedure were done according to the published approach [25]. In brief, the intensity of insulin staining and the area of islet were measured by two professional pathologists who were unware of the experiment being performed. The intensity of insulin staining was scored as 0 (absent), 1 (weak), 2 (moderate), 3 (strong), or 4 (very strong). And the results were shown by the ration between two groups.

### Transient Transfection and Luciferase Reporter Assay

NF-κB transcriptional activity was assayed in Min6 cells using the NF-κB luciferase reporter construct. A plasmid containing the β-galactosidase gene driven by the cytomegalovirus promoter (Clontech Laboratories, Palo Alto, CA, USA) was used as an internal control. Min6 cells grown in 24-well plates were transfected with plasmids containing the NF-κB luciferase reporter construct and β-galactosidase using the Lipofectamine Plus transfection kit, according to the manufacturer’s instructions (Invitrogen, CA, USA). Twenty-four hours after transfection, the cells were treated with or without uric acid for 6 h. After the cells were lysed using 1×passive lysis buffer, luciferase activity was determined as previously described [26].

### Cycloheximide Chase Assay

Min6 cells were transfected with MafA-overexpressing plasmid. After overnight culture, the cells were treated with 100 µg/ml cycloheximide and 5 mg/dL uric acid or normal medium, followed by lysis at the indicated time points and analysis of MafA protein levels by Western blotting.

### Statistical Analysis

Statistical analysis was performed with SPSS 11.0 software. Comparisons between groups were made using one-way ANOVA, followed by Student’s t-test. Results are presented as means ± SEM. Values of *P*<0.05 were considered to be significant.

## Results

### Administration of Uric Acid to Mice Results in an Abnormal Response to Glucose

**Figure 1:**
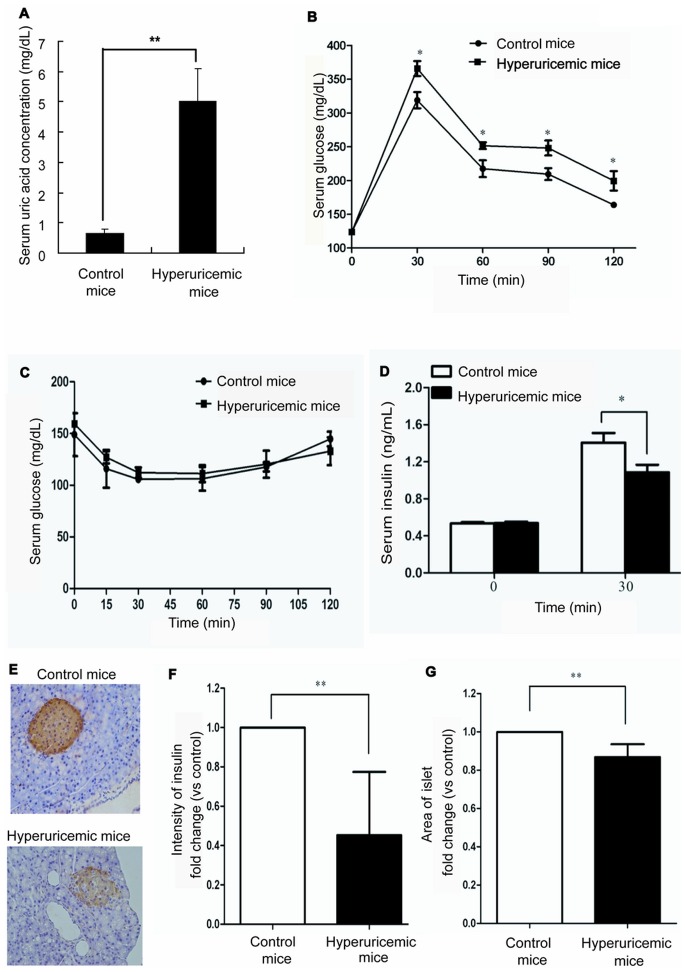
Administration of uric acid to mice results in an abnormal response to glucose. (**A**) Serum uric acid levels of mice were significantly increased after daily injection of uric acid (250 mg/kg) for 4 weeks. (**B**) This figure showed the results of glucose tolerance test. The glucose tolerance test (2 mg/kg) led to significantly elevated blood glucose levels in hyperuricemic mice compared to controls. (**C**) This figure showed the results of insulin tolerance test. In the insulin tolerance test (0.75 U/kg), there was no difference in blood glucose levels between hyperuricemic mice and control mice. (**D**) Plasma insulin level was significantly lower in hyperuricemic mice at 30 min than in the control group. (**E**) Immunohistology using antibody to insulin showed reduced insulin content in islets from hyperuricemic mice compared with that of control mice (original magnification,×200). (**F**) Fold changes in insulin content were determined in hyperuricemic mice and control mice. Insulin content was significantly reduced in hyperuricemic mice. (**G**) Fold changes in islet area were determined in hyperuricemic mice and control mice. Islet area was significantly reduced in hyperuricemic mice. *P<0.05, **P<0.01.

To demonstrate the effects of hyperuricemia on murine glucose metabolism, 8-week-old BALB/c mice were given daily intraperitoneal injections of uric acid (250 mg/kg) for 4 weeks, while control mice were injected with normal saline. Serum uric acid levels were monitored to evaluate the potency of the uric acid treatment regime. As shown in Fig. 1A, the serum uric acid levels of mice injected with uric acid (5.03±1.05 mg/dL) were significantly increased compared to those of the control mice (0.63±0.15 mg/dL). To assess glucose homeostasis in the hyperuricemic mice, intraperitoneal glucose tolerance (2 mg/kg) and insulin tolerance tests were performed. As shown in Fig. 1B, at time points 30, 60, 90, and 120 min after the start of the glucose tolerance test, blood glucose levels were significantly elevated in hyperuricemic mice compared with the controls. Meanwhile, mice in the hyperuricemic group exhibited significantly lower insulin levels at 30 min than mice in the control group (Fig. 1D). This effect was not considered to result from the development of insulin resistance, since uric acid treatment did not affect fasting glucose levels (Fig. 1B), or from peripheral insulin sensitivity, as demonstrated by insulin tolerance tests (Fig. 1C) in the hyperuricemic mice.

In addition to the observation of metabolic parameters, morphological analysis was performed to investigate the specific effects of uric acid on pancreatic islets. In comparison to control mice treated with vehicle alone, chronic uric acid treatment resulted in a reduction in both insulin content and islet area in the hyperuricemic pancreas (Fig. 1E–G; *P*<0.05).

### Uric Acid Activates the NF-κB Signaling Pathway in Pancreatic β-cells

Following exposure to damaging factors, including inflammatory cytokines, high glucose, and elevated free fatty acids, activation of the NF-κB signaling pathway has been reported as a key event promoting death and dysfunction of pancreatic islet cells [26]. Interestingly, uric acid has been observed to affect NF-κB signaling activation in endothelial cells and proximal tubule cells of the kidney [27], [28]. These observations led us to examine the possible involvement of NF-κB in uric acid-treated pancreatic β-cells.

**Figure 2:**
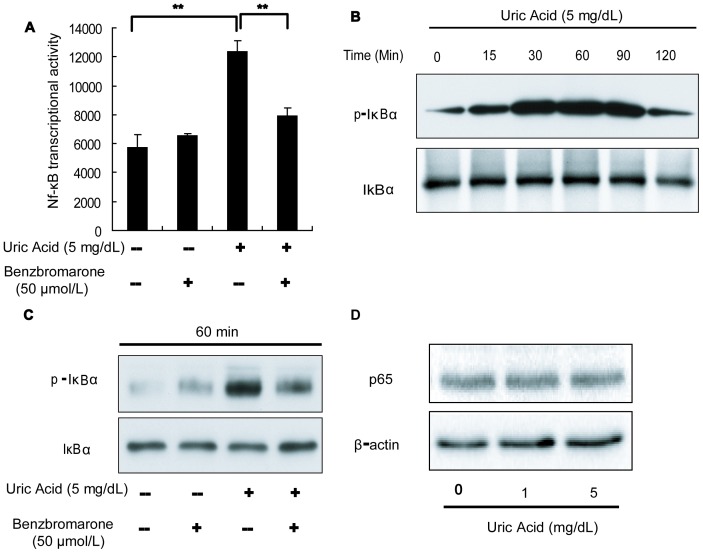
Uric acid activates the NF-κB signaling pathway in pancreatic β-cells. (**A**) Treatment of Min6 cells with 5 mg/dL uric acid for 24 h upregulated NF-κB transcriptional activity, as detected by a luciferase reporter assay. The uric acid-induced increase in NF-κB transcriptional activity was reversed by benzbromarone. (**B**) IκBα phosphorylation, detected by Western blotting, increased in a time-dependent manner in Min6 cells stimulated with 5 mg/dL uric acid at 60 mins. (**C**) Benzbromarone (50 µmol/L) reversed the uric acid-induced increase in IκB phosphorylation. (**D**) Total p65 protein levels in Min6 cells, detected by Western blotting, were unaffected by treatment with different concentrations of uric acid. **P<0.01.

Treatment of Min6 cells with 5 mg/dL uric acid for 24 h strongly upregulated NF-κB transcriptional activity (Fig. 2A). In addition, the effect of uric acid on NF-κB transcriptional activity was also significantly inhibited by 50 µmol/L benzbromarone – a specific inhibitor of uric acid transporters (Fig. 2A). The activation of NF-κB by uric acid was also monitored by observation of the phosphorylation of IκB, which leads to translocation of NF-κB from the cytoplasm to the nucleus and the initiation of NF-κB target gene expression. Western blot analysis showed that treatment of Min6 cells with 5 mg/dL uric acid markedly enhanced phosphorylation of IκBα in a time-dependent manner (Fig. 2B). The expression of phosphorylated protein peaked 60 min after treatment, followed by a slow decrease over time. It was interesting that 50 µmol/L benzbromarone attenuated the uric acid-induced IκBα phosphorylation by 60% (Fig. 2C). However, no difference in total p65 expression was detected between cells treated with vehicle alone or with uric acid (Fig. 2D). These data suggest that the administration of uric acid activates the NF-κB signaling pathway by enhancing IκBα phosphorylation.

### Uric Acid Causes Excessive Nitric Oxide Production in Pancreatic β-cells via the NF-κB Signaling Pathway

**Figure 3:**
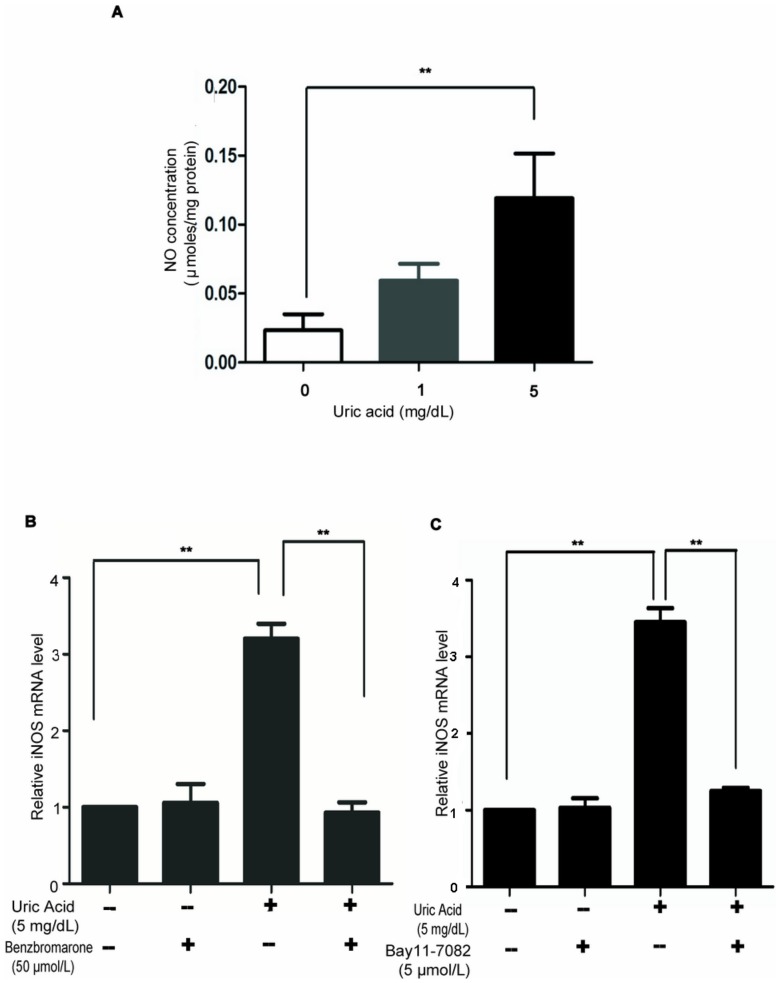
Uric acid causes excessive nitric oxide production in pancreatic β-cells via the NF-κB signaling pathway. (**A**) The NO content, determined by a Griess assay kit, of Min6 cells treated with different concentrations of uric acid. NO production increased in a concentration-dependent manner. (**B**) Uric acid treatment of Min6 cells induced an increase in iNOS mRNA levels, as detected by RT-PCR. This effect was reversed by 50 µmol/L benzbromarone. (**C**) 5 µmol/L BAY 11–7082 also reversed the uric acid-induced increase in iNOS mRNA levels. **P<0.01.

Excessive NO production caused by immunological and inflammatory stimulation is regarded as one of the important molecular mechanisms leading to apoptosis of pancreatic β-cells [29]. Inducible nitric oxide synthase (iNOS) has been confirmed as one of the downstream targets of the NF-κB pathway. Transcriptional activation of iNOS by NF-κB leads to production of large amounts of NO [30]. This led us to investigate the effect of uric acid on NO production in pancreatic β-cells. Min6 cells were treated with 0, 1, or 5 mg/dL uric acid and the production of NO in the form of nitrite was determined. As shown in Fig. 3A, incubation with 5 mg/dL uric acid for 24 hours yielded a significant increase in NO production compared with the vehicle control. The effect of uric acid on NO output was confirmed by an increase in iNOS mRNA expression following treatment with a high concentration of uric acid (5 mg/dL). Expression of iNOS mRNA was attenuated by benzbromarone (Fig. 3B) and also by the NF-kB inhibitor BAY 11–7082 (5 µmol/L) (Fig. 3C). This difference of iNOS expression was also detected in the the islets isolated from the hyperuricemic mice and control mice by Western Blot Assay (Fig. S1). These data indicate that the NO overproduction induced by uric acid was mediated by activation of the NF-κB signaling pathway.

### Uric Acid Impairs β-cell Viability by Inducing Apoptosis

**Figure 4:**
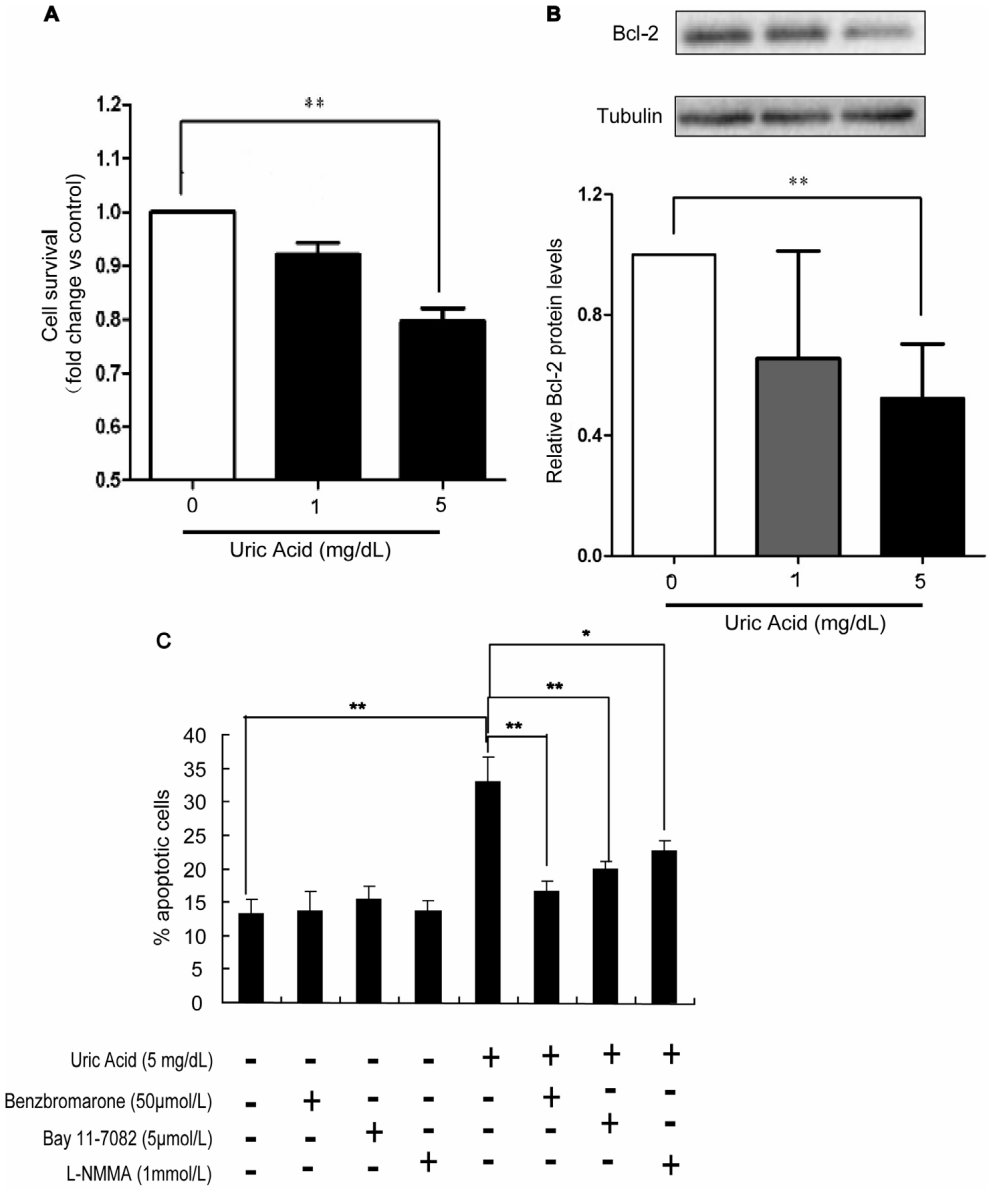
Uric acid impairs β-cell viability by inducing apoptosis. (**A**) The viability of Min6 cells was measured using an MTT assay after treatment with different concentrations of uric acid. Uric acid (5 mg/dL) significantly reduced cell survival. (**B**) Bcl-2 protein levels in Min6 cells treated with different concentrations of uric acid were determined by Western blotting. Bcl-2 expression relative to control levels was significantly reduced by uric acid treatment (5 mg/dL). (**C**) Min6 cells were treated with uric acid (5 mg/dL) for 48 h, followed by staining with TUNEL and Hoechst. Apoptosis was determined by scoring cells displaying pycnotic nuclei. A significant increase in apoptotic cells induced by uric acid was attenuated by 50 µmol/L benzbromarone, 5 µmol/L BAY 11–7082, and 1 mmol/L L-NMMA. About 2000 cells were scored for each group in one experiment. Values are means ± SEM and are representative of three separate experiments. *P<0.05, **P<0.01.

Since activation of the NF-κB signaling pathway and excessive NO production have been implicated in the impaired survival capacity of pancreatic β-cells, we investigated whether stimulation with uric acid affected β-cell viability. Min6 cells were treated with 0 (vehicle control), 1, or 5 mg/dL uric acid for 48 h. Cell viability was measured using an MTT assay. Stimulation of Min6 cells with uric acid (5 mg/dL) had a significant inhibitory effect on cell viability compared with the vehicle control (Fig. 4A). Moreover, Min6 cells treated with uric acid displayed reduced Bcl-2 protein expression in comparison with controls (Fig. 4B). The similar trend of Bcl-2 expression was also detected in the the islets isolated from the hyperuricemic mice and control mice by Western Blot Assay (Fig. S1). The effect of uric acid on apoptosis was also analyzed by scoring cells displaying pycnotic nuclei (visualized by staining with the DNA-binding dye, Hoechst 33342) and TUNEL assay. Apoptosis induced by uric acid treatment was significantly attenuated by benzbromarone, the NF-κB inhibitor BAY 11–7082, or the NOS inhibitor L-NMMA(1 mmol/L), indicating that activation of the NF-κB signaling pathway and consequent NO overproduction were involved in the impairment of β-cell viability by uric acid (Fig. 4C, Fig. S2).

### Uric Acid Treatment Decreases Glucose-stimulated Insulin Secretion

**Figure 5:**
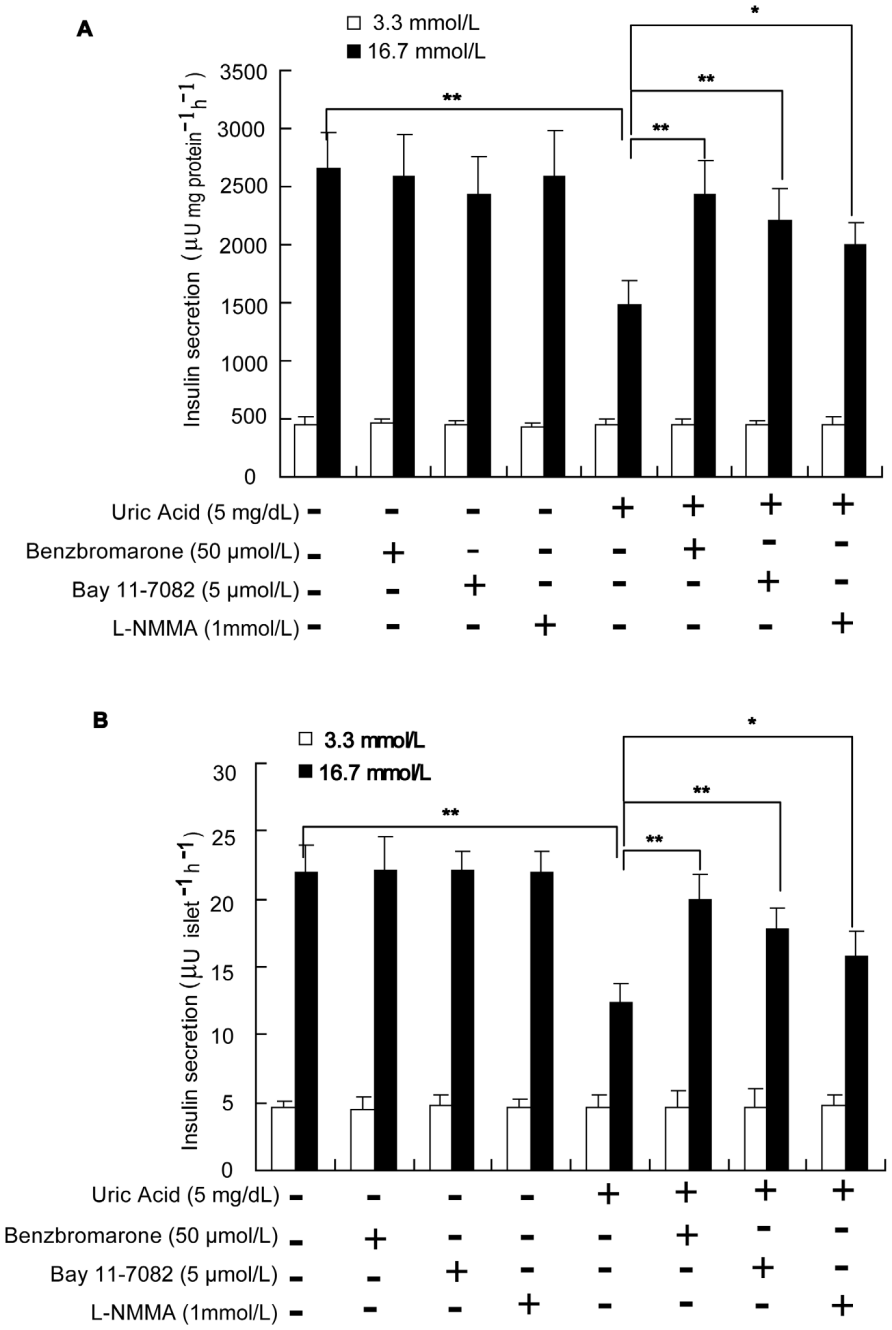
Uric acid treatment decreases glucose-stimulated insulin secretion. (**A**) Min6 cells were treated with uric acid (5 mg/dL), uric acid+benzbromarone (50 µmol/L), uric acid+BAY 11–7082 (5 µmol/L), or uric acid+L-NMMA (1 mmol/L) for 24 h. (**B**) Isolated mouse islets (eight islets per well) were treated as above. After incubation for 1 h in glucose-free KRB buffer, Min6 cells or mouse islets were treated for 1 h with KRB buffer containing low (3.3 mmol/L) or high (16.7 mmol/L) concentrations of glucose, and the supernatant fractions were collected for insulin concentration analysis. Uric acid treatment reduced glucose-stimulated insulin secretion by both Min6 cells and islets, and in both cases insulin secretion was restored by all three inhibitors. *P<0.05, **P<0.01.

Min6 cells and mouse islets were treated with 5 mg/dL uric acid for 24 h and challenged with different concentrations of glucose. Under baseline conditions of 3.3 mmol/L glucose, uric acid-treated β-cells secreted an equivalent amount of insulin to the controls. However, uric acid-treated Min6 cells and mouse islets demonstrated a 42% and 44% decrease in insulin secretion, respectively, compared to their controls when challenged with 16.7 mmol/L glucose (*P*<0.01; Fig. 5A, B).

Furthermore, the deleterious effect of uric acid on insulin secretion was significantly attenuated by benzbromarone, a specific inhibitor of uric acid transporters. To determine if NF-κB activation and consequent NO overproduction contributed to this uric acid-induced reduction in insulin secretion, we used the NF-κB inhibitor BAY 11–7082 or the iNOS inhibitor L-NMMA together with uric acid to treat Min6 cells and mouse islets. Insulin secretion following stimulation with 16.7 mmol/L glucose was significantly restored in uric acid-treated Min6 cells and islets by incubation with L-NMMA or BAY 11–7082 (Fig. 5A, B).

### Uric Acid Negatively Regulates MafA Expression and Function

**Figure 6:**
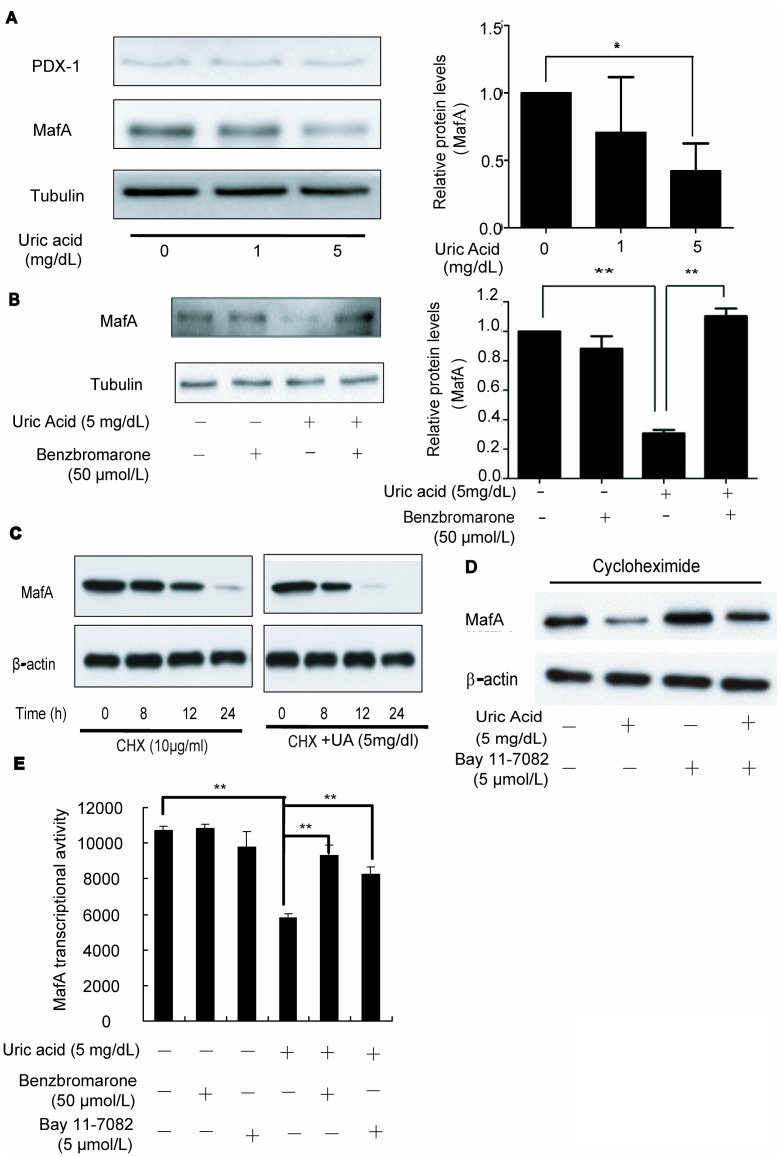
Uric acid negatively regulates MafA expression and function. (**A**) PDX-1 and MafA protein levels were determined by Western blotting in freshly isolated mouse islets treated with different concentrations of uric acid. Changes in expression levels of MafA relative to controls were also quantified. Expression of MafA but not PDX-1 was significantly reduced by uric acid (5 mg/dL). (**B**) MafA protein levels relative to controls were measured in Min6 cells treated with 5 mg/dL uric acid and 50 µmol/L benzbromarone. Benzbromarone significantly reversed the inhibitory effect of uric acid on MafA expression. (**C**) MafA protein levels were detected in Min6 cells treated with uric acid (UA) and the protein synthesis inhibitor cycloheximide (CHX, 10 µg/ml). MafA protein degraded more rapidly in the presence of uric acid than in controls. (**D**) MafA protein levels were detected in Min6 cells treated with 5 mg/dL uric acid, 10 µg/ml cycloheximide and 5 µmol/L BAY 11–7082. BAY 11–7082 partially reversed the suppressive effect of uric acid on MafA protein degradation. (**E**) The transcriptional activity of MafA was measured using a luciferase reporter assay in Min6 cells treated with uric acid (5 mg/dL), uric acid+benzbromarone (50 µmol/L), or uric acid+BAY 11–7082 (5 µmol/L). Uric acid reduced the transcriptional activity of MafA by 46%, and this effect was significantly attenuated by benzbromarone or BAY 11–7082. The results are expressed as a ratio of luciferase to β-galactosidase activity. *P<0.05, **P<0.01.

Insulin gene expression in adult β-cells is regulated by the glucose-responsive transcription factors PDX-1 and MafA amongst others. To examine whether uric acid impairment of insulin release involves these transcription factors, we measured the expression of these proteins in mouse islets cultured with different concentrations of uric acid by Western blotting. Treatment with 5 mg/dL uric acid for 12 h significantly suppressed MafA but not PDX-1 protein expression in isolated mouse islets (Fig. 6A). The similar change of MafA and PDX-1 expression was also observed in the the islets isolated from the hyperuricemic mice and control mice by Western Blot Assay (Fig. S1) The inhibitory effect of uric acid on MafA expression was also confirmed by Western blotting of uric acid-treated Min6 cells (Fig. 6B). Furthermore, the suppression of MafA expression by uric acid was significantly reversed by benzbromarone (Fig. 6B).

To determine whether uric acid downregulates MafA expression by enhancing its degradation, we treated Min6 cells with the protein synthesis inhibitor cycloheximide (CHX, 10 µg/ml) and measured the degradation rate of MafA in the presence and absence of uric acid. We found that MafA protein levels decreased more rapidly in the presence of uric acid than in its absence (Fig. 6C). Moreover, the NF-κB inhibitor BAY 11–7082 could partially reverse the suppressive effect of uric acid on MafA protein degradation (Fig. 6D).

To assess the effect of uric acid on the transcriptional activity of MafA on its target genes, we transfected Min6 cells with a luciferase reporter vector containing the rat *InsI* MafA recognition element (MARE). As shown in Fig. 6E, treatment with 5 mg/dL uric acid reduced the transcriptional activity of MafA by 46% in β-cells compared to controls treated with vehicle alone. Interestingly, the deleterious effect of uric acid on the transcriptional activity of MafA was significantly attenuated by benzbromarone or BAY 11–7082.

## Discussion

Hyperuricemia, characterized by an increased level of uric acid in the serum, has frequently been documented in individuals with metabolic syndrome. In recent years, multiple large clinical studies have been performed to explore the potential association of hyperuricemia and future risk of type 2 diabetes, since metabolic syndrome is an established risk factor for type 2 diabetes. A positive relationship between hyperuricemia and impaired glucose metabolism has been reported in several cross-sectional [1], [31], [32] and prospective studies [33], [34]. However, the underlying mechanism by which hyperuricemia is linked to an increased risk for diabetes is still unknown.

The current study provides evidence for the first time that uric acid has a direct impact on pancreatic β-cells through the NF-κB-iNOS-NO pathway. We report here that the treatment of normal mice with uric acid for 4 weeks markedly increased the serum uric acid level in the experimental animals, and this was accompanied by abnormally high levels of blood glucose due to the insufficient amount of insulin produced by β-cells in response to glucose challenge. In addition, an insulin tolerance test showed no difference between the uric acid-treated mice and control animals, indicating that the effects of uric acid on glucose metabolism were not related to insulin resistance. We therefore attribute these effects directly to the action of uric acid on pancreatic β-cells. In support of this hypothesis, isolated mouse islets treated with uric acid secreted significantly less insulin in response to glucose stimulation than did the islets treated with vehicle alone. Furthermore, this impaired capacity of the β-cells to secrete insulin was significantly restored by the addition of benzbromarone, an inhibitor of uric acid transporters, to the culture medium of uric acid-treated islets. It should be mentioned that the question of whether elevated levels of uric acid impair insulin sensitivity still remains controversial. Both insulin resistance and β-cell failure are regarded as two key events of type 2 diabetes development. Some studies have shown that insulin sensitivity is significantly lower in hyperuricemic subjects, suggesting that hyperuricemia causes type 2 diabetes maybe through impaired insulin sensitivity [35], [36], [37]. Interestingly, some other researchers have demonstrated that the uric acid levels of hyperuricemic patients have no effect on their insulin sensitivity index [38]. Even in a study linking insulin resistance with hyperuricemia, Simental-Mendía et al. reported the failure of β-cell function to compensate for variation of the insulin sensitivity in hyperuricemic individuals with impaired glucose tolerance [39]. Their results also supported the hypothesis that hyperuricemia may have a direct negative effect on pancreatic β-cells. Since administration of uric acid for 4 weeks did not result in any apparent effect on the insulin sensitivity of the experimental animals in the current investigation, a further in vivo study with longer periods of uric acid treatment may be needed to clarify the association of hyperuricemia and insulin sensitivity.

The activation of the NF-κB-iNOS-NO signaling pathway has been reported to lead to the cytotoxicity and apoptosis in β-cells in both type 1 and type 2 diabetes [40], [41]. In unstimulated cells, the NF-κB heterodimers are retained in the cytoplasm by a family of inhibitors, called IκBs. The best-studied and most important member of this family is IκBα. Activation of the classical pathway of NF-κB involves the phosphorylation and subsequent degradation of IκBα, leading to the release and translocation of NF-κB to the nucleus. This enables NF-κB to turn on the expression of specific downstream genes with DNA-binding sites for NF-κB, causing the given physiological or pathological responses [42]. Our studies show for the first time that in mouse pancreatic β-cells, uric acid caused phosphorylation of IκB and subsequent activation of the NF-κB signaling pathway in pancreatic islets, leading to an inflammatory effect. This observation is in agreement with previous reports showing that uric acid activates the NF-κB signaling pathway in mononuclear cells, smooth muscle cells, and rabbit proximal tubule cells [23], [27], [43]. iNOS is one of the major downstream genes of the NF-κB pathway. Based on our observations of iNOS expression and NO production in pancreatic β-cells stimulated by uric acid, it is reasonable to speculate that expression of the iNOS gene was induced by uric acid, at least in part, through the NF-κB signaling pathway. This supposition is supported by further data demonstrating that the uric acid transporter inhibitor benzbromarone and the NF-κB inhibitor BAY 11–7082 reduced uric acid-induced iNOS gene expression in Min6 cells. These data suggest the involvement of the NF-κB-iNOS-NO pathway in uric acid-induced β-cell suppression. Indeed, in further experiments we found that uric acid treatment resulted in the presence of markedly increased numbers of apoptotic Min6 cells. Meanwhile, inhibition of either the NF-κB pathway by BAY 11–7082 or of iNOS expression by L-NNMA rendered the β-cells significantly resistant to uric acid-induced apoptosis.

In addition to causing β-cell death, we found that treatment of isolated mouse islets with uric acid also resulted in a significant decrease in insulin production. This impaired β-cell function was partially restored by the uric acid transporter inhibitor benzbromarone and NOS inhibitor. Next we asked the question, how does uric acid impair glucose-stimulated insulin secretion in β-cells? Our results demonstrated that the expression of MafA was significantly decreased by uric acid in a dose-dependent manner. It is of interest that uric acid reduced insulin production through repression of MafA (Fig. 6A), but not other major insulin transcription factors, such as PDX-1 or NeuroD (data not shown). It has become evident that MafA degradation through the proteasomal system leads to defective insulin secretion by β-cells [44], [45]. Meanwhile, a growing body of evidence suggests that nitric oxide is able to induce the degradation of various proteins through different mechanisms [14]. Here we demonstrate that uric acid treatment enhanced MafA degradation in Min6 cells. Moreover, MafA stability could be restored by inhibition of NF-κB activity. The precise mechanism by which nitric oxide enhances MafA degradation in the uric acid-treated β-cell will be further explored in future studies.

The normal serum uric acid level in rodents is approximately 1 mg/dL. By referring to relevant published studies [15], [46], we chose for our *in vitro* studies the uric acid concentrations of 1 and 5 mg/dL, which were also observed to be within the *in vivo* range of uric acid levels in our hyperuricemic mice. However, we need to emphasize that although the protective effects of inhibition of the NF-κB-iNOS-NO axis and uric acid uptake by β-cells *in vitro* have been clearly demonstrated, it remains to be determined whether this therapeutic strategy is feasible *in vivo* for preventing β-cell injury. In addition to pancreatic β-cell, other endocrinological cells in islet, e.g. alpha cells, delta cells, are confirmed relevant to the development of type 2 diabetes as well. It is very intriguing to observe the impacts of uric acid on the other endocrinology cells in pancreatic islets in addition to beta cell.

In conclusion, we have found that soluble uric acid can directly cause β-cell death and dysfunction by activation of the NF-κB-iNOS-NO signal axis. The underlying molecular mechanism reported in our study provides further support for previous epidemiological findings regarding the involvement of uric acid in the pathogenesis of type 2 diabetes. It is worth speculating that controlling the serum uric acid level in hyperuricemic patients may prevent the incidence of type 2 diabetes in this particular patient population.

## Supporting Information

Figure S1 **Decreased expression of MafA and Bcl-2, but increased expression of iNOS were detected in the islets isolated from hyperuricemic mice.** Islets were isolated from both the hyperuricemic mice and the control mice. The expression of MafA, PDX-1, iNOS and Bcl-2 proteins in the islets was detected using Western Blotting. Compared to the islets from control mice, the islets isolated from hyperuricemic mice demonstrated the downregulated expression of MafA and Bcl-2 but upregulated iNOS expression. There was no difference of PDX-1 expression observed between the islets obtained from the hyperuricemic and control mice.
(TIF)

Figure S2 **The uric acid induced apoptosis of pancreatic b cell was attenuated by the inhibitors of urate transporter, iNOS and NF-κB signaling pathway.** Apoptosis of β-cells was observed by TUNEL Assay (original magnification, 400×). The cultured INS-1 cells were divided into the following groups: (A) Control; (B) Bay11–7082 (5 µmol/L); (C) L-NMMA (1 mmol/L); (D) Benzbromarone (50 µmol/L); (E) Uric acid (5 mg/dL); (F) Uric acid (5 mg/dL) + Bay11–7082 (5 µmol/L); (G) Uric acid (5 mg/dL) + L-NMMA (1 mmol/L); (H) Uric acid (5 mg/dL) + Benzbromarone (50 µmol/L). TUNEL positive cells were shown in green fluorescence. Apoptosis induced by uric acid treatment was significantly attenuated by the urate transporter inhibitor benzbromarone, the NF-κB inhibitor BAY 11–7082, or the iNOS inhibitor L-NMMA (1 mmol/L).
(TIF)
