# Supplementary figures and images for: Hyperuricemia Causes Pancreatic β-Cell Death and Dysfunction through NF-κB Signaling Pathway
Source: PLoS One. 2013 Oct 25;8(10):e78284. doi: 10.1371/journal.pone.0078284 (PMC3808354; doi:10.1371/journal.pone.0078284)

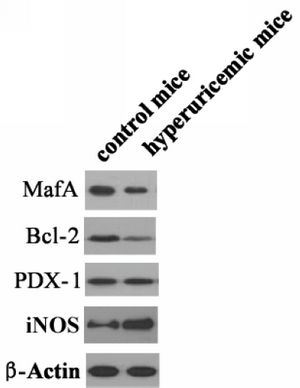

Supplement: Figure S1 — Decreased expression of MafA and Bcl-2, but increased expression of iNOS were detected in the islets isolated from hyperuricemic mice. Islets were isolated from both the hyperuricemic mice and the control mice. The expression of MafA, PDX-1, iNOS and Bcl-2 proteins in the islets was detected using Western Blotting. Compared to the islets from control mice, the islets isolated from hyperuricemic mice demonstrated the downregulated expression of MafA and Bcl-2 but upregulated iNOS expression. There was no difference of PDX-1 expression observed between the islets obtained from the hyperuricemic and control mice. (TIF) [file pone.0078284.s001.tif]

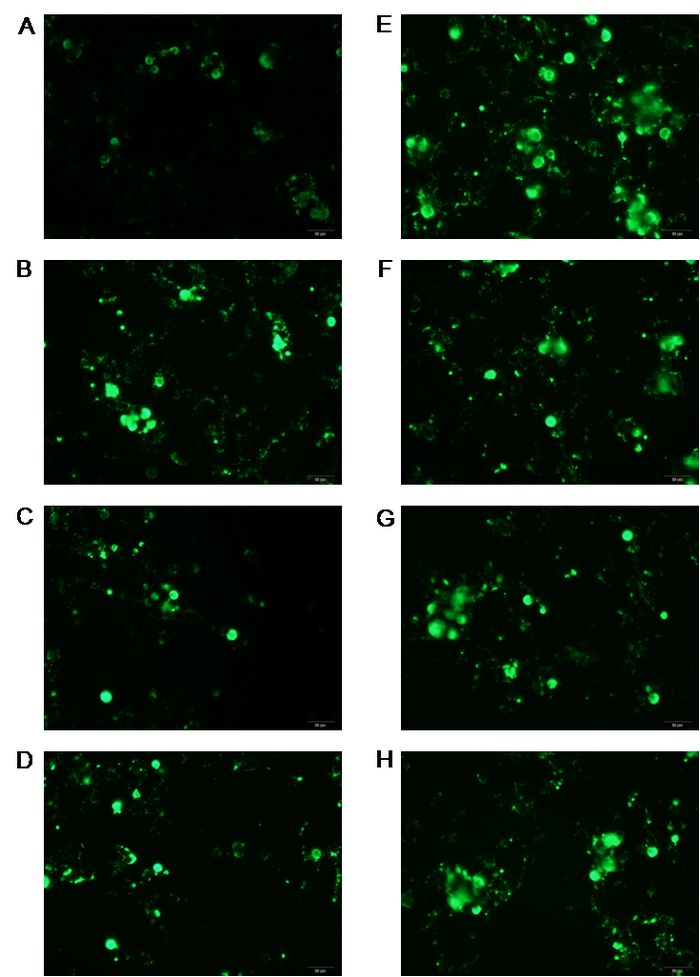

Supplement: Figure S2 — The uric acid induced apoptosis of pancreatic b cell was attenuated by the inhibitors of urate transporter, iNOS and NF-κB signaling pathway. Apoptosis of β-cells was observed by TUNEL Assay (original magnification, 400×). The cultured INS-1 cells were divided into the following groups: (A) Control; (B) Bay11–7082 (5 µmol/L); (C) L-NMMA (1 mmol/L); (D) Benzbromarone (50 µmol/L); (E) Uric acid (5 mg/dL); (F) Uric acid (5 mg/dL) + Bay11–7082 (5 µmol/L); (G) Uric acid (5 mg/dL) + L-NMMA (1 mmol/L); (H) Uric acid (5 mg/dL) + Benzbromarone (50 µmol/L). TUNEL positive cells were shown in green fluorescence. Apoptosis induced by uric acid treatment was significantly attenuated by the urate transporter inhibitor benzbromarone, the NF-κB inhibitor BAY 11–7082, or the iNOS inhibitor L-NMMA (1 mmol/L). (TIF) [file pone.0078284.s002.tif]
